# Dysregulation of protein succinylation and disease development

**DOI:** 10.3389/fmolb.2024.1407505

**Published:** 2024-05-31

**Authors:** Xiaoli Hou, Lijuan Zhu, Haiying Xu, Jie Shi, Shaoping Ji

**Affiliations:** ^1^ Center for Molecular Medicine, Zhengzhou Shuqing Medical College, Zhengzhou, Henan, China; ^2^ Zhengzhou Central Hospital Affiliated to Zhengzhou University, Zhengzhou, Henan, China; ^3^ Zhoukou Vocational and Technical College, Zhoukou, Henan, China; ^4^ Department of Biochemistry and Molecular Biology, Medical School, Henan University, Kaifeng, Henan, China

**Keywords:** post-translational modification, succinylation, dysregulation, disease, succinylase

## Abstract

As a novel post-translational modification of proteins, succinylation is widely present in both prokaryotes and eukaryotes. By regulating protein translocation and activity, particularly involved in regulation of gene expression, succinylation actively participates in diverse biological processes such as cell proliferation, differentiation and metabolism. Dysregulation of succinylation is closely related to many diseases. Consequently, it has increasingly attracted attention from basic and clinical researchers. For a thorough understanding of succinylation dysregulation and its implications for disease development, such as inflammation, tumors, cardiovascular and neurological diseases, this paper provides a comprehensive review of the research progress on abnormal succinylation. This understanding of association of dysregulation of succinylation with pathological processes will provide valuable directions for disease prevention/treatment strategies as well as drug development.

## 1 Introduction

Protein posttranslational modification **(**PTM) encompasses the covalent processing that proteins undergo after translation, involving many biological processes (Wu et al., 2022). At present, the most common PTMs are phosphorylation, acetylation and succinylation, etc (Mu et al., 2021).

**FIGURE 1:**
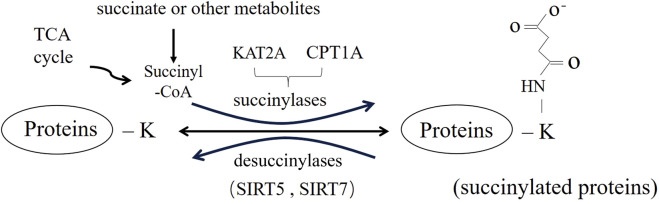
Reversible modification of lysine succinylation and its regulatory factors.

Lysine succinylation (Ksucc) is a new, broad-spectrum, dynamic, reversible PTM that has been discovered in recent years (Figure 1) (Zhang H. et al., 2023). It is involved in almost all biological processes of organisms (Ye and Li, 2022), playing an important role in the metabolic regulation, signal transduction and cell differentiations (Amirkashani et al., 2023; Huang et al., 2023), mainly through the regulation of protease activity and gene expression (Huang et al., 2021). The regulation of succinylation and involves multiple factors, including succinyl-donor and the regulators of succinylation (Lu and Han, 2022). Dysregulation of succinylation, changing activity and aberrant function of protein involved in energy metabolism and downstream epigenetic modification (Chinopoulos, 2021), is closely related to the occurrence and development of diseases such as inflammation, tumors and others (Zhang et al., 2022). In this review, we intend to discuss the occurrence of succinylation dysregulation, including the characteristics, distribution and mechanism of the dysregulation. Meanwhile, we will summarize the theoretical and experimental evidence of succinylation dysregulation in various diseases, triggering further research on succinylation as a future therapeutic target for diseases.

## 2 Occurrence of succinylation

Succinylation is a process by which a negatively charged four-carbon succinyl group is covalently bound to the primary amine of lysine residue by enzymatic or non-enzymatic means (Ali et al., 2020). It leads to significant changes in protein structure due to the large spatial structure of the succinyl group, as well as a change in charge from +1 to −1 for the lysine residue, resulting in alterations in physicochemical properties and biological functions of proteins (Dai et al., 2022). Succinylases such as CPT1A and KAT2A are required in enzymatic succinylation, but sufficient succinyl-CoA supply is a pre-request for the occurrence of non-enzymic succinylation (Mu et al., 2021).

Succinylation is widely distributed in mitochondria and sub-cells (Zhang H. et al., 2023). In mitochondria, the abundance of succinyl-CoA is one of the main governing factors of Ksucc. The TCA cycle generates large amounts of succinyl-CoA, which may contribute to passive succinylation (Sreedhar et al., 2020). Secondly, an electrostatic attraction between succinyl CoA and lysine residues may play an auxiliary, but the detail mechanisms remained further study. Succinylation can be involved in differentiation, metabolism and other important life processing by regulating protease activity and gene expression (Ye and Li, 2022). This makes succinylation highly valuable for the study of diseases related to mitochondrial disorders (Yang Y. H. et al., 2022). For example, succinylation at K311 of glutaminase (GLS-K311succ) enhances its activity, offsetting oxidative stress while promoting tumor cell survival and growth pancreatic ductal adenocarcinoma (PDAC) (Tong et al., 2021). In nucleus, succinylation is present in over one-third of nucleosomes (histone/non-histone). H3-K79succ leads to increased β-catenin stability and subsequently promotes gene expression of cyclin D1, c-Myc, GLUT1 and lactate dehydrogenase A (LDHA) in tumorigenesis (Wang et al., 2018). FEN1 K77succ reduced the accumulation of DNA damage and sensitivity to fork stalling agents through enhancing its bond with Rad9-Rad1-Hus1 (Shi et al., 2020). Succinylation directly regulates genome-wide transcription and DNA repair activity through chromatin remodeling (Zorro Shahidian et al., 2021).

## 3 Regulation and dysregulation of succinylation

Normally, succinylation is strictly regulated by the succinyl-donor, succinylases, and desuccinylase (Lu and Han, 2022). Changes of the concentration of succinyl-donors and activity of enzymes can cause dysregulation of succinylation (Lu et al., 2021).

### 3.1 Influence of the succinyl-donor on succinylation

Succinyl-CoA serves as the primary donor for succinylation, and its concentration reversibly regulates the process of succinylation (Chinopoulos, 2021). Overall decrease in the mitochondrial succinyl-CoA pool, decreased myofibril protein succinylation, which may promote heart failure (Ali et al., 2020). Succinyl-CoA deficiency, impeding ketone oxidation in skeletal muscle, is often associated with acute episodes of ketoacidosis (Mechchate et al., 2023). Enzymes affecting succinyl-CoA production indirectly regulate succinylation levels. For instance, α-ketoglutaric dehydrogenase (α-KGDH) promotes succinylation by increased succinyl-CoA produced in propionate and/or ketone body metabolism in nerve cell, accelerating progression of neurodegenerative diseases (Dobolyi et al., 2020).

Additionally, succinate or other metabolites can also impact the extent of succinylation (Guillon et al., 2022). Accumulation of ischemic succinate increased the succinylation of the Rho family GTPase Cdc42, resulting in neural stem cell proliferation inhibition and aggravated cerebral ischemia/reperfusion (I/R) injury (Huang et al., 2023). Therefore, targeting on succinylation is considered as a potential therapeutic approach for I/R (Liu et al., 2020).

### 3.2 Effects of succinylases on succinylation

In recent years, significant progresses have been made in the study of succinylases, with the identification of several new succinylases, such as lysine acyltransferase 2A (KAT2A), and carnitine palmitoyltransferase 1A (CPT1A) (Lu et al., 2021).

KAT2A is also known as General Control Non-derepressible 5 (GCN5), whose histone succinylases activity plays a crucial role in tumorigenesis (Tong et al., 2020). KAT2A-mediated H3-K79succ regulates gene expression and β-catenin stability in tumor cells, contributing to tumor cell proliferation and invasion (Tong et al., 2021). In prostate cancer (PCa) cells, KAT2A-mediated CTBP- K46 and K280succ can repress transcription suppressing activity of it, thus acting as an oncogene (Zhou et al., 2023).

CPT1A has also been identified as a potential succinylases. In gastric cancer (GC), CPT1A-mediated LDHA-K222succ reduces its binding to SQSTM1 and inhibits the degradation of LDHA, as well as promotes GC invasion and proliferation.

### 3.3 Effects of desuccinylases on succinylation level

Sirtuin5(SIRT5) possess NAD^+^-dependent desuccinylation activity (Green and Storey, 2021; Zhang and Goetzman, 2021), reducing succinylation levels of mitochondrial proteins (Chinopoulos, 2021), and subsequently modulating the target activities of numerous substrate proteins to maintain metabolic homeostasis (Lukey et al., 2020). SIRT5 protein reduces LDHA-K118succ in PCa, by which it may be used as a new strategy to prevent the progression of castration-resistant PCa for treatment (Kwon et al., 2023). SIRT5 can inhibit peroxisom-induced oxidative stress, liver protection and inhibit the development of hepatocellular carcinoma by reducing the succinylation level of peroxisomal ACOX1 (Chen et al., 2018). Consequently, SIRT5 is considered a pivotal regulator in various cancers and inhibitors targeted succinylation may serve as promising anti-tumor (Shen et al., 2023).

Additionally, it has been discovered that SIRT7 possesses desuccinylase activity, primarily in nucleus (Bai et al., 2022). It serves important functions such as stimulating the expression of ribosomal RNA, facilitating DNA damage repair, and balancing chromatin compaction (Lagunas-Rangel, 2023). These findings emphasize the critical role of SIRT7 in protecting chromatin structure, controlling innate immune regulation and ensuring reproductive protection during stem cell senescence (Raza et al., 2024).

## 4 Dysregulation of succinylation and diseases

Dysregulation of succinylation plays a crucial role in the occurrence and progression of diseases since activity/sublocation of disease-related proteins or key enzymes are changed (Dai et al., 2022). Therefore, the relationship between dysregulation of succinylation and diseases can provide theoretical support for disease treatment and related drugs (Liu et al., 2022) (Table 1).

**TABLE 1:** Locations, influence and regulatory factors of Ksucc proteins in diseases.

Diseases		Ksucc proteins	Ksucc sites	Influence	Regulatory factors	References
Inflammation and Tuberculosis	fungal infections	Aconitase in *S. erythraea* HL3168 E3	K278	Inhibit metabolism in the host	erythromycin	Ke et al. (2022)
			K373			
	Tuberculosis	EchA19 in *Mtb*	K132		succinyl-CoA	Bonds et al. (2020)
			K139			
	Hepatitis B	histone H3	K79	Promote the replication of HBV	GCN5	Yuan et al. (2020b)
	influenza pneumonia	Nucleoprotein influenza virus	K87	Disrupt the influenza replication cycle	succinate	Guillon et al. (2022)
Tumor	PDAC	histone H3	K79 K311	promote proliferation, migration and invasion of tumor cells	KAT2A SIRT3-5	(Tong et al., 2020; Tong et al., 2021)
		GLS				
	HCC	PGAM1	K99		Aspirin	Wang et al. (2023)
		hRIDA	K293		SIRT5	Siculella et al. (2021)
	GC	S100A10	K47		CPT1A	Wang et al. (2022)
		FBN1	k672			Li et al. (2020)
		LDHA	K222			
	gliomas	TAGLN2	K40		—	Zhang et al. (2023b)
	PCa	CTBP1	K46		KAT2A	Zhou et al. (2023)
			K280			
		LDHA	K118		SIRT5	Kwon et al. (2023)
	colon cancer	CS	K393	Inhibit the proliferation and migration of cancer cells		Ren et al. (2020)
			K395			
	breast cancer and lung cancer	GLS	K164			Lukey et al. (2020)
			K311			
			K158			
cardiovascular diseases	hypertrophic cardiomyopathy	ECHA	—	promote myocardial fibrosis, reduce cardiac function	SIRT5	Sadhukhan et al. (2016)
	heart failure	IDH2				Chang et al. (2021)
	TAA、TAD	PKM、LDHA、SDHA				Zhang et al. (2023a)
neurological diseases	AD	APP and tau protein	—	promote the development of AD	SIRT5	Yang et al. (2022b)
	I/R	GTPase Cdc42		aggravate cerebral I/R injury		Huang et al. (2023)
	anxiety	PDHC		decrease parasympathetic activity and anxiety indicators		Artiukhov et al. (2022)

### 4.1 Dysregulation of succinylation and inflammation/tuberculosis

As mentioned above, protein succinylation, as a conservative PTM, participates in diverse biological processes in bacteria, fungi, viruses and human cells (Yuan T. et al., 2020).

Succinylation plays an important role in protein biosynthesis and carbon metabolism of bacteria/fungi and antibiotic biosynthesis. The succinylation levels of vancomycin-intermediate *Staphylococcus aureus* (*VISA*) decreased with enhanced vancomycin tolerance (Yang et al., 2024). EchA19-K132, 139succ in *Mycobacterium tuberculosis* (*Mtb*) is a negative feedback regulator of cholesterol metabolism in the host (Bonds et al., 2020). In addition, increased succinylation exerting favorable inhibition of *Aspergillus fumigatus* infection in terms of fungicidal and enhanced macrophage killing effect (Chen et al., 2023). These findings provide valuable insights into the mechanism of desuccinylase inhibitors in the treatment of ITR-resistant fungal infections.

Succinylation dysregulation may play a critical role in viral infection. SARS-CoV-2, the pathogen responsible for the COVID-19 pandemic, induces succinylation of several crucial enzymes in the tricarboxylic acid cycle (TCA), leading to inhibition of cellular metabolic pathways (Liu et al., 2022). Notably, IFN-α clears hepatitis B virus (HBV) cccDNA through depressed GCN5-mediated H3-K79succ (Yuan Y. et al., 2020). Therefore, inhibitors targeting succinylation exhibit significant antiviral effects, providing provide a new avenue for anti-viral treatment.

### 4.2 Dysregulation of succinylation and immunity/tumor

Dysregulation of succinylation affects the infiltration of immune cells and the expression of immune genes, thereby promoting the malignant development of cancer (Lu et al., 2021). Succinylation induces the expression of pro-inflammatory genes in T cells and activates HIFα in M1 macrophages, leading to the inflammation-cancer cycle (Shen et al., 2023).

Succinylation is tissue heterogeneous during tumorigenesis (Lu and Han, 2022), and it is involved in the regulation of various tumorigenesis and progression through different substrate targets or signaling pathways (Zhang X. et al., 2023).

In general, succinylation can have a pro-cancer effect. It is significantly high-expressed in various tumor tissues such as lung cancer, PCa, HCC, PDAC and glioma (Zhang X. et al., 2023; Zhou et al., 2023). Elevated succinylation is closely related to tumor invasion/metastasis ability and patient survival prognosis (Lukey et al., 2020). There are two mechanisms for the pro-cancer effect of succinylation. One involves inhibiting degradation of succinylated substrate, thus promoting proliferation, invasion and migration of tumor cells. Upregulation of CPT1A and downregulation of SIRT5 synergistically promotes S100A10-K47succ and fibrillin 1 (FBN1) K672succ, resulting in the accumulation of S100A10 and FBN1 in GC cells and further promoting tumor progression (Wang et al., 2022). Another mechanism is that succinylation helps maintain the redox balance of cancer cells and promotes their proliferation. In colon cancer, elevated PKM2-K498succ promotes the production of more ATP during glucose starvation and maintains tumor cell survival during nutrient depletion (Qi et al., 2019).

It is paradoxical that high citrate synthase (CS) K393 and 395succ significantly inhibits the proliferation and migration of colon cancer cells (Ren et al., 2020), and the detection of increased SIRT5 expression levels in lung cancer and breast cancer (Lu and Han, 2022). This suggests that increased succinylation contributes to the suppression of certain tumors.

### 4.3 Dysregulation of succinylation in cardiovascular diseases

The concentration of succinyl-CoA in the heart tissue is significantly higher than that in any other organs. Succinylation is closely associated with cardiomyocyte metabolism and its dysregulation is widely involved in cardiovascular disease (CVD) (Weis et al., 2022). Highly succinated proteins highly enriched in thoracic aortic aneurysms (TAA) and thoracic aortic dissection (TAD) Promise to be potential diagnostic markers and therapeutic targets for aortic diseases (Zhang H. et al., 2023). Accumulation of succinylated ECHA leads to decreased ATP production in the myocardium, reduced cardiac ejection fraction, and ultimately to hypertrophic cardiomyopathy (Hershberger et al., 2018). During myocardial ischemia, SIRT5 significantly promotes the desuccinylation of IDH2, a key enzyme involved in TCA, maintaining mitochondrial homeostasis and improving myocardial fibrosis, reduce the incidence of heart failure (Chang et al., 2021).

### 4.4 Dysregulation of succinylation in neurological diseases

The analysis of succinylated proteomics data and transcriptomics data revealed that the mRNAs matched by most differentially succinylated proteins were especially highly expressed in neurons and astrocytes (Deng et al., 2021). Abnormal succinylation may be linked to abnormal cortical nerve anatomy and could potentially contribute to the pathological processes of various neurological disorders (Ning et al., 2022). In Alzheimer’s disease, increased succinylation level of amyloid precursor protein disrupts its normal proteolytic processing, leading to the accumulation of Aβ and plaque formation. Additionally, succinylation of tau protein promotes its aggregation into tangles and impairs microtubule assembly (Yang Y. et al., 2022). An elevated succinylation level of pyruvate dehydrogenase complex (PDHC) in the hippocampus may downregulate mitochondrial energy metabolism, while potentially contributing to parasympathetic activity dysregulation, anxiety and depression (Artiukhov et al., 2022). Therefore, studying the mechanism and function of desuccinylation can provide a foundation for targeted therapy of nervous system diseases.

### 4.5 Dysregulation of succinylation in other diseases

Dysregulation of succinylation is associated with reproductive disorders (Yang et al., 2018). Reduction of LDHC-K317succ reduces production of ATP, leading to asthenospermia (Yang et al., 2020). Inhibition succinylation of germ cell can result in reproductive injury. Analysis of postmenopausal women aged from 55 to 70 years old indicate that succinylation of apolipoprotein A-I and A-II, hemoglobin subunit α and haptoglobin are elevated in patients with osteoporosis and osteopenia (Zhang et al., 2019). This suggests that elevated succinylation is associated with aging and age-related diseases. (Du et al., 2018).

## 5 Perspective

It is well-known that succinylation and its dysregulation are closely related to the processes in cells, including variety of physiological and pathological processes. Although dysregulation of succinylation has been extensively investigated in regulation of metabolism and epigenetics, the factors underlying variations in succinylation levels across different tumors or other conditions remain to be fully elucidated. It is necessary for scientists to identify accurate roles of succinylases and succinyl-CoA in executing succinylation on different molecules, distinguishing active and passive modification of succinylation.

As for disease, succinylation modification is mainly involved in metabolism regulation, particularly in energy production, thus obesity and type II diabetes are most likely to relate to dysregulation of succinylation. Further and extensive investigations are required to explore its roles and accurate mechanism in trigger those diseases. In addition, a variety of PTMs usually act in combination, such as high overlap between succinylation and acetylation (Ye and Li, 2022), and the interaction between them also needs to be further researched. It is apparent that there could be competition among various modifications occurring on the lysine residue, given that lysine is the most frequently modified residue in post-translational modifications.

Along with the development of proteomics research with mass spectrum technology, the in-depth study of the above issues will help us more accurately understand the relationship of succinylation dysregulation and disease development, and provide a theoretical foundation for treatment of the diseases and the development of related drugs.
